# Analysis of the long-term solubility and phases of five different root canal sealers in phosphate buffered saline: an *in vitro* study

**DOI:** 10.7717/peerj.20853

**Published:** 2026-02-19

**Authors:** Bridhi Jalan, Karthik Shetty, Vasavi Kumblekar, Roma M, Heeresh Shetty

**Affiliations:** 1 Department of Conservative Dentistry and Endodontics, Manipal College of Dental Sciences Mangalore, Manipal Academy of Higher Education, Manipal, India; 2 Department of Biochemistry, Kasturba Medical College, Mangalore, Manipal Academy of Higher Education, Manipal, Karnataka, India; 3 Department of Conservative Dentistry and Endodontics, Nair Hospital Dental College, Mumbai, Maharashtra, India

**Keywords:** Root canal sealers, Solubility, AH Plus, AH Plus bioceramic, Crystallinity, MTA Fillapex

## Abstract

**Background:**

The long-term success of root canal therapy depends substantially on the physicochemical stability of endodontic sealers, particularly their solubility and the development of stable crystalline phases during setting. Tricalcium silicate-based sealers (TSBSs) have shown promising biological properties, yet their reported solubility profiles remain inconsistent, raising concerns regarding degradation, gap formation, and the potential for periapical irritation. This study evaluated and compared the solubility and crystalline phase evolution of five commercially available root canal sealers immersed in phosphate-buffered saline (PBS) over extended time intervals.

**Methods:**

Twenty-five disc-shaped specimens (*n* = 5 per material) were prepared using AH Plus Bioceramic (AHPBC), AH Plus (AHP), Ceraseal (CS), BioRoot RCS (BRCS), and MTA Fillapex (MTAF). Solubility was assessed gravimetrically after immersion in PBS for 24 h, 28 days, and 6 months. Phase analysis was performed at each interval using X-ray diffraction (XRD) to identify changes in crystallinity.

**Results:**

The solubility values varied markedly among the materials and increased over time for most groups. AHP consistently demonstrated the lowest solubility across all intervals, whereas MTAF and BRCS presented the highest values. XRD analysis revealed distinct crystalline phase patterns for each sealer. AHPBC and CS maintained relatively stable crystalline structures throughout the study period. In contrast, BRCS and MTAF exhibited noticeable fluctuations, and AHP showed high crystallinity initially and after 6 months, indicating greater structural stability.

**Conclusion:**

AHP displayed the most favourable physicochemical stability, with minimal solubility and sustained crystallinity. AHPBC and CS also demonstrated stable long-term behaviour, whereas BRCS and MTAF showed greater solubility and structural changes. These findings suggest limitations in the long-term durability of these materials. These findings underscore the importance of sealer selection, particularly when considering long-term stability and the potential impact of material dissolution within the root canal system.

## Introduction

Endodontic sealers remain central to the long-term success of non-surgical root canal therapy, forming a thin but decisive interface that complements the core filling and helps prevent reinfection. The ability of these teeth to seal accessory canals, adhere to dentin, entomb residual microorganisms, and retain dimensional stability directly influences treatment outcomes (Komabayashi et al., 2020; Cimpean et al., 2022). Although modern materials have advanced considerably, the principles laid down by Grossman—low solubility, adequate radiopacity, biological compatibility, and resistance to disintegration—continue to guide present-day expectations from any sealer used in clinical practice (Gopikrishna, 2021).

Among contemporary materials, tricalcium silicate-based sealers (TSBSs) have gained considerable interest due to their bioactivity, ion-release profile, and potential to promote interfacial hydroxyapatite formation (Margono et al., 2025). However, their solubility behaviour remains one of the most contentious aspects of their performance. Reports documenting inconsistent dissolution patterns under different experimental conditions highlight the possibility of interfacial voids, micro-gaps, and subsequent bacterial leakage if solubility becomes excessive (El-Mansy, Ali & Hassan, 2020; Jasrasaria, Tikku & Bharti, 2023; Katakidis et al., 2025). Similarly, crystallinity plays an equally important role, as well-formed hydration products and mature crystalline phases help the material withstand degradation and maintain the integrity of the seal over time (Saber et al., 2023). Understanding how both features evolve during prolonged exposure is therefore essential when assessing the long-term reliability of a sealer.

Since their introduction in 2007, premixed flowable TSBSs formulations have gained popularity for their convenience and uniformity (Dong & Xu, 2023). AH Plus Bioceramic (AHPBC) is a relatively new representative of this category, composed of zirconium dioxide, tricalcium silicate, and lithium carbonate, and is designed to offer good handling while limiting calcium ion release in the absence of dicalcium silicate (Raman & Camilleri, 2024). Ceraseal (CS), another premixed TSBS, incorporates tricalcium and dicalcium silicates with tricalcium aluminate and is manufactured to provide consistent sealing ability (Saber et al., 2023). MTA Fillapex (MTAF), although often grouped with bioceramic sealers, contains a salicylate resin base with only a modest proportion of MTA; this combination has been linked to incomplete hydration and greater dimensional and chemical instability (Komabayashi et al., 2020; Ashkar et al., 2023). BioRoot RCS (BRCS), supplied as a powder-liquid hydraulic calcium silicate cement, has been shown to stimulate periodontal ligament fibroblasts, modulate inflammation, and support hard-tissue formation (Jeanneau et al., 2019; Wuersching et al., 2022). In contrast, the epoxy-amine resin sealer AH Plus (AHP) remains the benchmark because of its low solubility, strong adhesion, and dependable dimensional stability (De Souza et al., 2023).

For *in vitro* assessment, phosphate-buffered saline (PBS) is now widely acknowledged as a more representative medium than distilled water, since it allows a closer approximation of clinical conditions and offers insight into how sealers behave when exposed to physiological ionic environments (Moraes et al., 2022). While a number of studies have examined selected physicochemical properties of individual sealers, major gaps persist. Only one recent investigation has reported medium-term solubility data for AHPBC (Donnermeyer et al., 2022), and long-term (6-month) solubility and crystallinity assessments of multiple sealers—including AHPBC—remain unavailable.

In this context, the present study evaluated the solubility by mass loss and crystalline phase behaviour of five different sealers representing distinct chemical classes: premixed TSBS (AHPBC and CS), powder-liquid TSBS (BRCS), salicylate resin-based MTAF, and the epoxy-resin-based AHP. All the materials were immersed in PBS for 24 h, 28 days, and 6 months. The null hypothesis stated that no significant differences would be observed in solubility or crystalline phase characteristics among the five evaluated sealers over the selected time intervals.

## Materials and Methods

### Study design and ethical considerations

This *in vitro* study evaluated the solubility by gravimetric method and crystalline phase characteristics of five commercially available root canal sealers. As no human or animal samples were involved, ethical approval was not needed; however, permission from the Institutional Scientific Committee was obtained (Protocol Reference No. 23052).

### Sample size determination

A total of 25 samples were prepared, with five samples allocated to each sealer group (*n* = 5). The sample size was calculated to achieve 90% statistical power with an alpha error of 1%, ensuring adequate sensitivity to detect differences among the materials.

### Materials tested

Five root canal sealers representing distinct chemical formulations were included: AH Plus Bioceramic (AHPBC), AH Plus (AHP), Ceraseal (CS), BioRoot RCS (BRCS), and MTA Fillapex (MTAF). Their compositions and manufacturer details are summarized in Table 1.

**Table 1:** Composition of the five root canal sealers assessed.

Sealer	Manufacturer	Composition
AH Plus (AHP)	Dentsply Sirona, Charlotte, NC, USA	**Paste A**: diepoxide, calcium tungstate, zirconium oxide, aerosol, and pigment. **Paste B**: 1-adamantane amine; N, N’-dibenzyl-5- oxa-nonandiamine-1,9; tricyclodecane diamine; calcium tungstate; zirconium oxide; aerosol; and silicone oil
AH Plus Bioceramic (AHPBC)	Dentsply Sirona, Charlotte, NC, USA	Zirconium dioxide, tricalcium silicate, dimethyl sulfoxide, lithium carbonate, and thickening agents
MTA Fillapex (MTAF)	Angelus, Londrina, Parana, Brazil	**Base paste** (yellow): salicylate resin, natural resin, calcium tungstate, nanoparticulated silica, pigments. **Catalyst paste** (white): diluting resin, mineral trioxide aggregate (MTA), nanoparticulated silica, pigments.
Bioroot RCS (BRCS)	Septodont, Saint Maur-des-Fosses, France	**Powder**: tricalcium silicate, zirconium oxide and povidone. **Liquid**: aqueous solution of calcium chloride and polycarboxylate.
Ceraseal (CS)	MetaBiomed, South Korea	Zirconium dioxide, tricalcium silicate, dicalcium silicate, tricalcium aluminate, thickening agents, polyethylene glycol (PEG)

### Solubility assessment

#### Preparation of PBS immersion medium

PBS was prepared with sodium chloride (80 g), potassium chloride (2 g), dipotassium hydrogen phosphate (2.4 g), and disodium phosphate (14.4 g), dissolved in distilled water. The pH was adjusted to 7.4 using hydrochloric acid, and the final volume was standardized to one L. PBS was used instead of distilled water to simulate better conditions encountered *in vivo*.

#### Specimen preparation

The disc-shaped samples were fabricated following ISO 6876:2012 recommendations. Teflon molds (1.5 ± 0.1 mm height; 7.75 ± 0.1 mm internal diameter) were cleaned with acetone for 1 min before use. Each sealer was prepared according to the manufacturer’s instructions and carefully packed into the molds on a glass plate, ensuring the absence of air voids.

The samples were allowed to set undisturbed at 37 °C for 24 h. AHP, AHPBC, and CS were stored under 95% relative humidity to ensure complete setting, while AHP and MTAF were set without added humidity. After setting, excess material was removed from the mold surface using 600-grit silicon carbide paper.

### Initial weight measurement (W_1_)

Each specimen was removed from its mold and weighed three times using a precision balance (accuracy: 0.001 g). The mean value was recorded as the initial mass (W_1_).

### Immersion protocol

The samples were suspended individually in 20 mL of fresh PBS at 37 °C using nylon threads, ensuring full exposure of all surfaces. After the first 24 h, the PBS was replaced weekly for the duration of the 6-month experimental period. The samples were stored in airtight containers at 100% relative humidity to prevent dehydration.

### Post-immersion weight measurement (W_2_)

At each time point (24 h, 28 days, and 6 months), the samples were removed, rinsed with three mL of distilled water, and dried in an incubator at 37 °C for 24 h. Each sample was weighed three times, and the mean value was recorded as the final mass (W_2_).

### Solubility calculation

The solubility (%) was calculated using the following formula:

\begin{eqnarray*}\mathrm{Solubility}= \frac{{W}_{1}-{W}_{2}}{{W}_{1}} \times 100 \end{eqnarray*}

where

W_1_ = initial mass

W_2_ = final mass after immersion.

### X-ray diffraction analysis

#### Sample preparation for XRD

For each sealer, the five samples from the solubility analysis were combined and ground using an agate mortar and pestle to obtain a fine, homogeneous powder. From this mixture, 0.2 g of powder per group was mounted between two layers of magic tape for XRD evaluation. As the initial samples were pooled, and X-ray diffraction (XRD) analysis was performed on one representative sample per material.

#### XRD procedure

Phase analysis was carried out using a continuous-scan X-ray diffractometer operating at 40 kV and 30 mA. Scans were recorded over a 2*θ* range of 0°–60° at a rate of 4°/min. Crystalline phases and relative crystallinity were quantified at 24 h, 28 days, and 6 months.

### Statistical analysis

The data were analysed *via* SPSS version 26.0 (IBM, Armonk, NY, USA). Normality of the distribution was assessed using the Shapiro–Wilk test. For within-group comparisons across time points, paired *t*-tests were performed. Differences among the sealer groups were evaluated using one-way ANOVA followed by Tukey’s *post hoc* test, which was used to identify specific pairwise differences. Statistical significance was set at *p* < 0.005.

## Results

### Solubility analysis

The mean solubility values for each material at the three evaluation intervals are summarized in Table 2. Distinct patterns emerged among the five sealers.

**Table 2:** Mean (%) ± standard deviation (SD) values of solubility for different types of sealer when immersed in phosphate-buffered saline.

**Time**	**AH PLUS BC**	**BIOROOT RCS**	**CERASEAL**	**MTA FILLAPEX**	**AH PLUS**	*p* value
After 24 h	3.49 ± 1.38	7.03 ± 1.93	3.23 ± 1.49	30.17 ± 21.69	1.37 ± 1.33	0.004
After 28 days	4.34 ± 1.89	7.06 ± 5.67	10.93 ± 3.26	16.06 ± 2.36	1.32 ± 1.07	0.001
After 6 months	5.402 ± 2.16	20.52 ± 2.48	15.51 ± 6.34	18.82 ± 8.11	0.83 ± 0.86	0.001

**Notes.**

*p* value <0.005 was set as statistically significant.

At 24 h, MTAF exhibited the highest solubility (30.17 ±  21.69%), followed by BRCS and AHPBC. CS showed a comparatively lower solubility (3.23 ± 1.49%), while AHP demonstrated the lowest value (1.37 ± 1.33%).

By 28 days, MTAF continued to have the greatest solubility (16.06 ± 2.36%). CS also exhibited increase in solubility (10.93 ± 3.26%), with BRCS and AHPBC presenting intermediate values. AHP again had the lowest solubility (1.32 ± 1.07%).

After 6 months, BRCS recorded the highest solubility (20.52 ±  2.48%), followed by MTAF (18.82 ± 8.11%). CS and AHPBC demonstrated moderate increases, and AHP maintained the lowest solubility (0.83 ± 0.86%).

Tukey’s *post hoc* analysis (Table 3) confirmed statistically significant differences across multiple comparisons. At 24 h, MTAF exhibited significantly greater solubility than AHPBC, CS, and AHP (*p* = 0.003, *p* = 0.003, and *p* = 0.001, respectively). Similar trends persisted at 28 days, where MTAF differed significantly from AHPBC (*p* = 0.001), BRCS (*p* = 0.002), and AHP (*p* = 0.001), and CS also significantly differed from AHP (*p* = 0.001). At 6 months, BRCS demonstrated significantly greater solubility than AHP did (*p* = 0.001), and MTAF differed significantly from both AHPBC (*p* = 0.002) and AHP (*p* = 0.001). Overall, the solubility increased significantly over time for most sealers, with the most pronounced changes observed in MTAF and BRCS.

**Table 3:** Tukey’s *post hoc* test for analysis of each of the root canal sealers in different time frames.

Variable	Comparison of	Comparison with	Mean difference	Standard deviation	*p* value
Percentage change after 24 h	AH PLUS BC	BIOROOT RCS	−3.53	6.20	0.753
		CERASEAL	0.26	6.20	1.000
		MTA FILLAPEX	−26.68	6.20	0.003
		AH PLUS	2.13	6.20	0.997
	BIOROOT RCS	CERASEAL	3.79	6.20	0.972
		MTA FILLAPEX	−23.15	6.20	0.010
		AH PLUS	5.66	6.20	0.889
	CERASEAL	MTA FILLAPEX	−26.94	6.20	0.003
		AH PLUS	1.87	6.20	0.998
	MTA FILLAPEX	AH PLUS	28.81	6.20	0.001
Percentage change after 28 days	AH PLUS BC	BIOROOT RCS	−2.72	2.06	1.000
		CERASEAL	−6.59	2.06	0.033
		MTA FILLAPEX	−11.72	2.06	0.001
		AH PLUS	3.02	2.06	0.595
	BIOROOT RCS	CERASEAL	−3.87	2.06	0.360
		MTA FILLAPEX	−9.00	2.06	0.002
		AH PLUS	5.74	2.06	0.075
	CERASEAL	MTA FILLAPEX	−5.13	2.06	0.132
		AH PLUS	9.61	2.06	0.001
	MTA FILLAPEX	AH PLUS	14.74	2.06	0.001
Percentage change after 6 months	AH PLUS BC	BIOROOT RCS	−15.11	3.07	0.981
		CERASEAL	−10.10	3.07	0.027
		MTA FILLAPEX	−13.42	3.07	0.002
		AH PLUS	4.58	3.07	0.579
	BIOROOT RCS	CERASEAL	5.01	3.07	0.494
		MTA FILLAPEX	1.69	3.07	0.980
		AH PLUS	19.69	3.07	0.001
	CERASEAL	MTA FILLAPEX	−3.32	3.07	0.814
		AH PLUS	14.68	3.07	0.001
	MTA FILLAPEX	AH PLUS	18.00	3.07	0.001

**Notes.**

*p* value <0.005 was set as statistically significant.

### Phase (XRD) analysis

**Figure 1:**
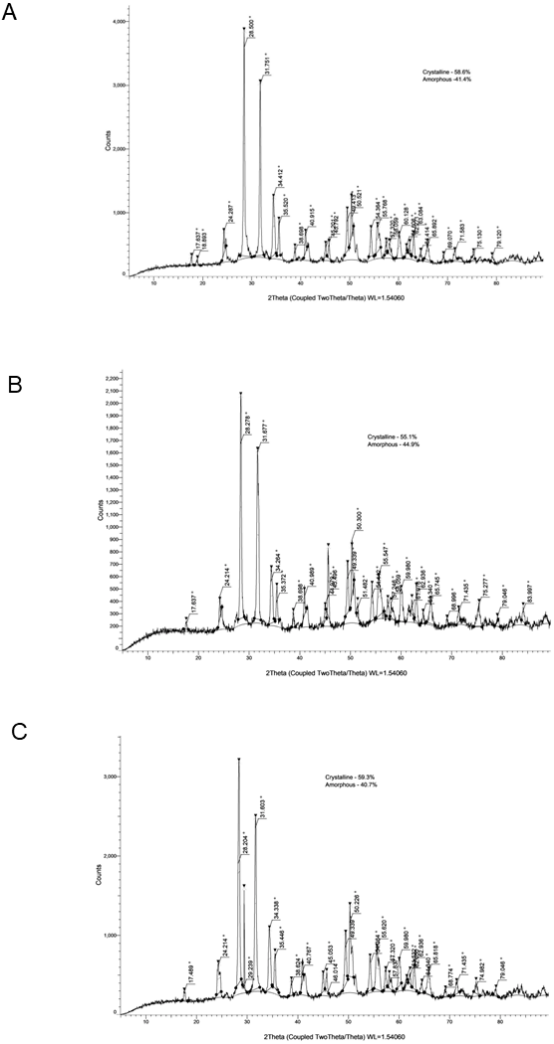
XRD analysis of AHPBC at 24 h (A), 28 days (B) and 6 months (C).

**Figure 2:**
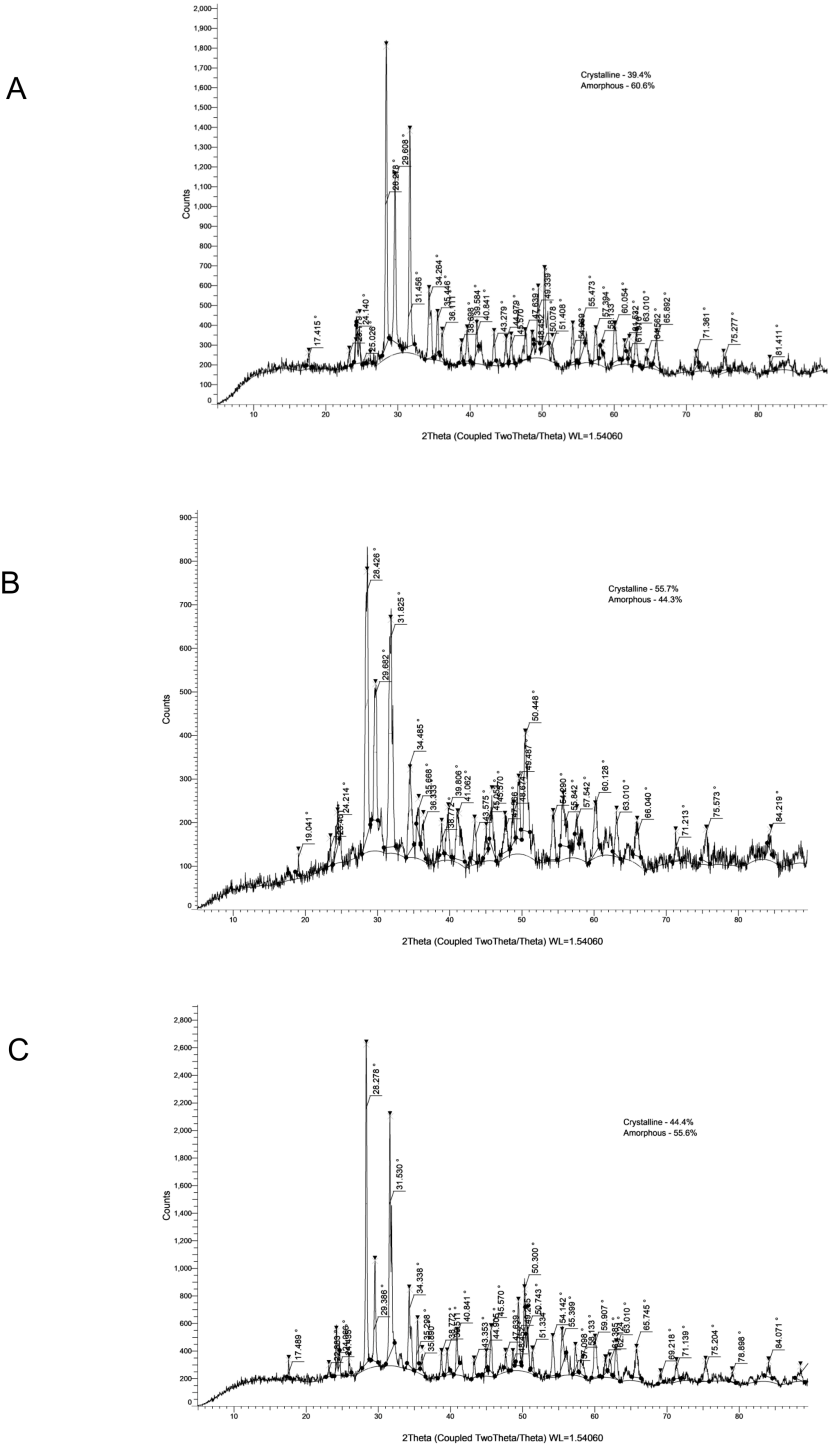
XRD analysis of BRCS at 24 h (A), 28 days (B) and 6 months (C).

**Figure 3:**
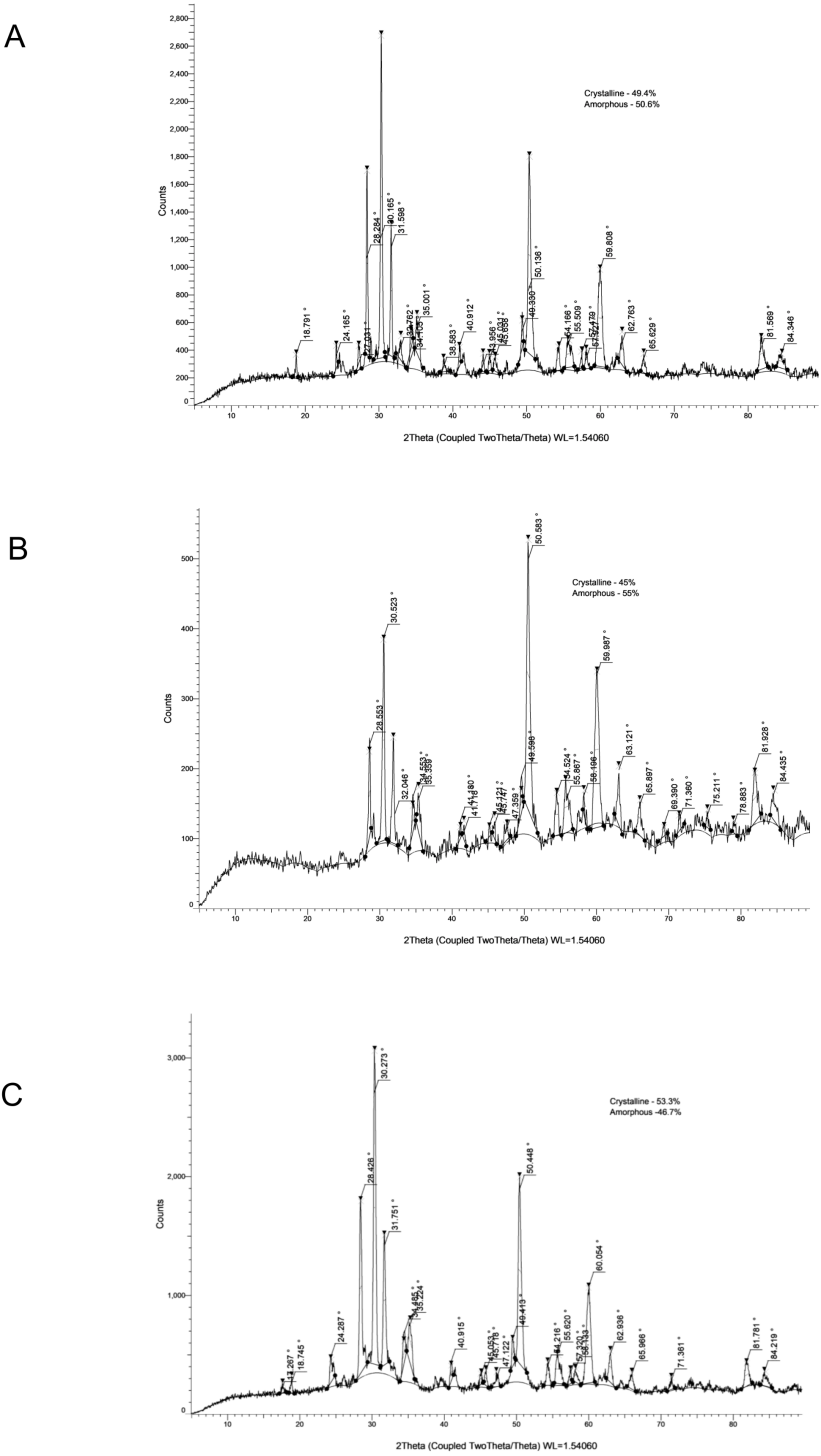
XRD analysis of Ceraseal at 24 h (A), 28 days (B) and 6 months (C).

**Figure 4:**
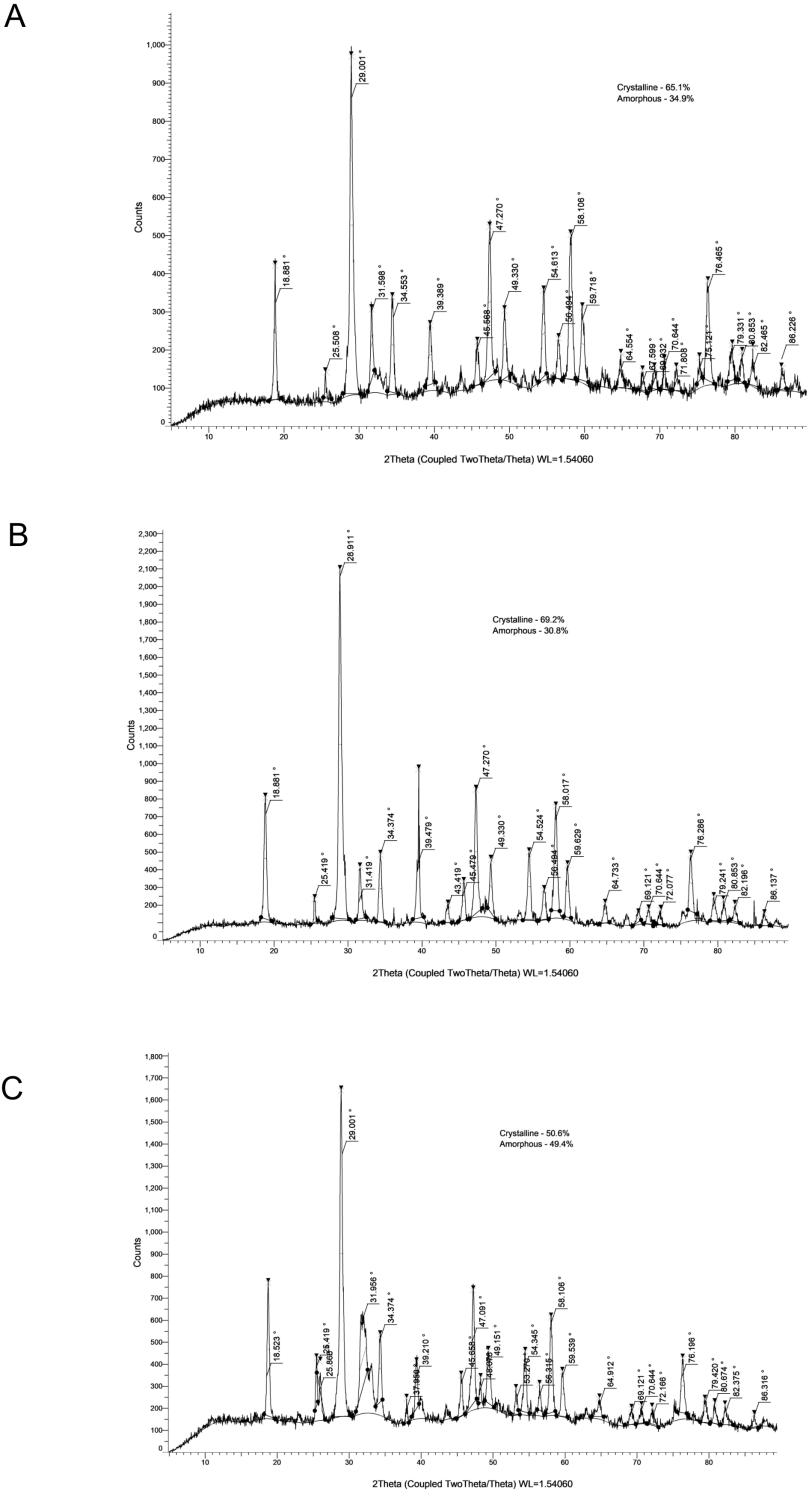
XRD analysis of MTAF at 24 h (A), 28 days (B) and 6 months (C).

**Figure 5:**
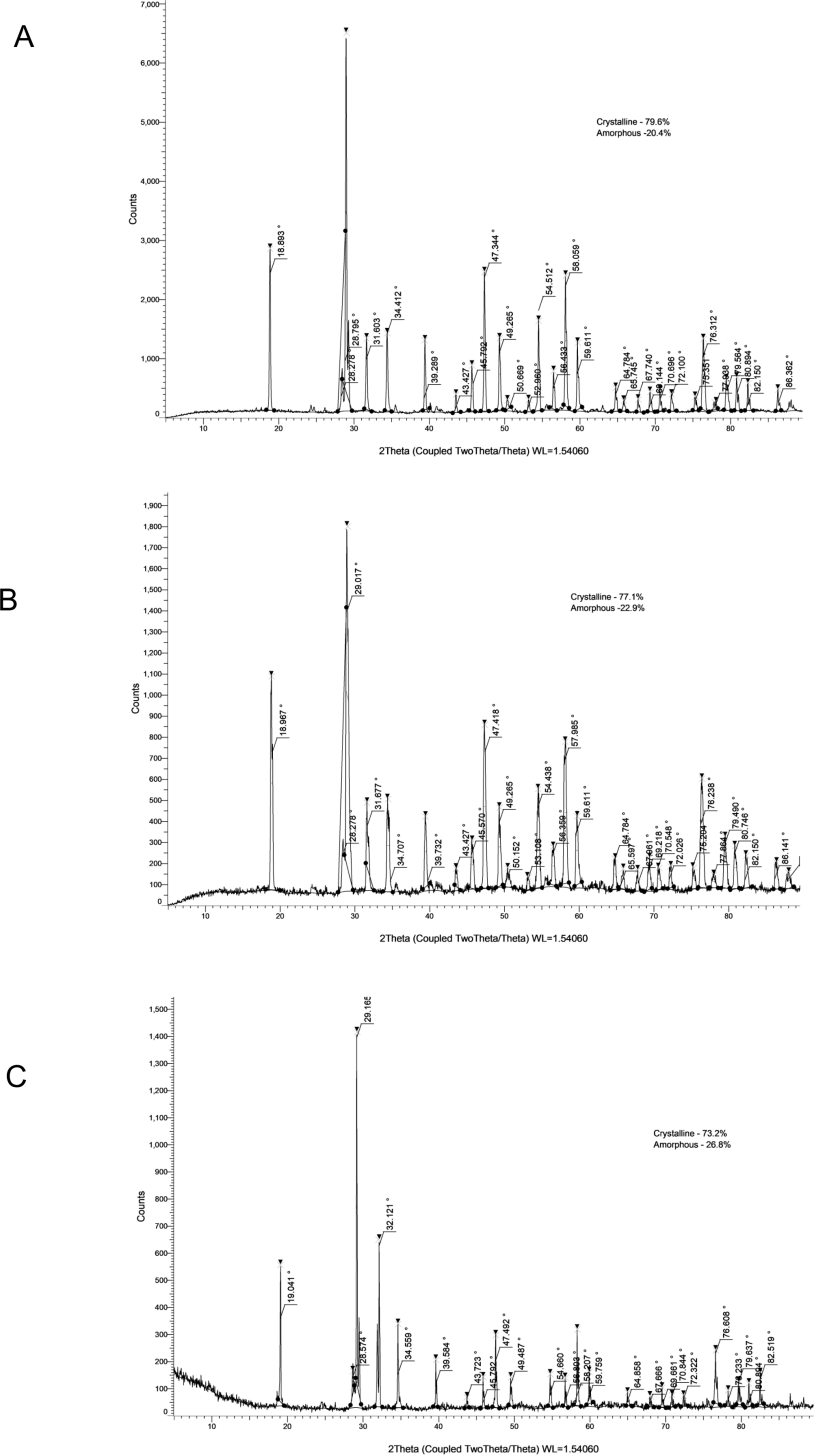
XRD analysis of AHP at 24 h (A), 28 days (B) and 6 months (C).

The crystallinity values and corresponding XRD patterns at each time point are presented in Table 4 and Figs. 1–5.

**Table 4:** Crystallinity (%) of different root canal sealers at 24 h, 28 days and 6 months.

**Groups**	**Crystallinity (%)**		
	**24 h**	**28 days**	**6 months**
AH PLUS BC	58.60	55.10	59.30
BIOROOT RCS	39.40	55.70	44.40
CERASEAL	49.40	45.00	53.30
MTA FILLAPEX	65.10	69.20	50.60
AH PLUS	79.60	55.10	73.20

### AHPBC

AHPBC showed a crystallinity of 58.60% at 24 h, which decreased slightly to 55.10% at 28 days and then increased to 59.30% at 6 months. The major diffraction peaks corresponded to zirconium oxide (monoclinic phase), hafnium oxide, calcium silicate hydrate, and calcium hydroxide [Ca (OH)_2_].

### BRCS

BRCS exhibited 39.40% crystallinity at 24 h, which rose to 55.70% at 28 days before decreasing to 44.40% at 6 months. The peaks identified included those of zirconium oxide (monoclinic phase), calcium silicate hydrate, and calcium hydroxide, reflecting hydration-related changes over time.

### CS

CS demonstrated crystallinity values of 49.40% at 24 h, 45.00% at 28 days, and 53.30% at 6 months. The identified phases included zirconium oxide, calcium silicate hydrate, and calcium hydroxide, indicating a relatively stable crystalline profile with mild fluctuations.

### MTAF

The MTAF presented a high initial crystallinity (65.10%), which increased to 69.20% at 28 days and then declined markedly to 50.60% at 6 months. XRD patterns revealed the presence of bismuth oxide, calcium hydroxide, calcium silicate hydrate, and calcium aluminate, suggesting changes associated with incomplete hydration and later phase instability.

### AHP

AHP demonstrated the highest degree of crystallinity among all sealers: 79.60% at 24 h, decreasing to 55.10% at 28 days, and increasing again to 73.20% at 6 months. Peaks corresponding to calcium tungstate and zirconium oxide were consistently observed, reflecting the predictable and stable setting characteristics of this epoxy-resin-based material.

#### Overall observations

Across all groups, Ca (OH)_2_ peaks were detectable, confirming active hydration processes and crystalline phase development characteristic of calcium silicate–based materials. AHP maintained the most stable structural profile throughout the evaluation period, whereas BRCS and MTAF demonstrated pronounced variability both in solubility and crystallinity, particularly at later intervals. These findings align with the solubility trends and underscore the differing long-term behaviours of the evaluated sealers.

## Discussion

This study examined the solubility and crystalline phase behaviour of five commercially available root canal sealers over a 6-month period in PBS. The findings revealed clear differences in the long-term physicochemical stability of these materials, with notable implications for their clinical performance. Although recent investigations have increasingly adopted micro–computed tomography (micro-CT) to quantify volumetric loss (Cavenago et al., 2014; Zancan et al., 2016; Pereira et al., 2019), the present study relied on the gravimetric method recommended by ISO 6876 and ANSI/ADA Specification No. 57. This standardized approach allowed reliable comparison with established literature and ensures reproducibility under controlled laboratory conditions.

Overall, the solubility values varied considerably among the sealers. MTA Fillapex (MTAF) exhibited the highest solubility at all time points, with a particularly pronounced mass loss within the first 24 h. Similar findings have been reported previously (Torres et al., 2019; Abu Zeid et al., 2021), and several material-specific factors may account for this behaviour. MTAF contains a salicylate resin matrix with a relatively low proportion of MTA, which may lead to incomplete hydration and an imbalanced polymerization reaction (Prado et al., 2018). The inclusion of bismuth oxide and certain resin components can further compromise setting and stability, contributing to increased leaching and higher solubility (Leme & Salvadori, 2022). Consistent with earlier reports, the present results suggest that the formulation of MTAF predisposes it to long-term dimensional instability.

BRCS also demonstrated a gradual increase in solubility over time, reaching its highest values at the 6-month interval. This pattern mirrors findings from previous investigations (El-Mansy, Ali & Hassan, 2020; Wuersching et al., 2022; Katakidis et al., 2025). Although BRCS is known for favorable biological responses and calcium ion release, the high solubility observed in the present study indicates continuous dissolution or surface degradation. This may be attributed to the fine hydrophilic particle size of TSBS, which increases the surface area of the fluid interaction (De Souza et al., 2023), as well as persistent release of Ca^2^^+^ and OH^−^ ions, which—while contributing to bioactivity—may also accelerate structural breakdown (Ziada et al., 2023).

CS and AHPBC displayed moderate solubility levels. Among the tested tricalcium silicate-based sealers, AHPBC had the lowest solubility. Although AHPBC did not satisfy the strict ISO criterion of <3% solubility at 24 h, its overall stability across the study period was superior to that of BRCS and MTAF. The relatively stable behaviour of these premixed materials may reflect the influence of zirconium oxide, which acts as a bioinert filler and contributes to maintaining the material’s structural framework (Zamparini et al., 2022; Hamdy et al., 2024).

Among all the materials tested, AHP consistently demonstrated the lowest solubility and the highest degree of structural stability. The highly cross-linked polymer network formed by the reaction between polyamine hardeners and epoxy resin is well known for its dimensional stability and resistance to hydrolytic degradation (Silva et al., 2021). The present findings reaffirmed the longstanding position of AHP as a benchmark sealer in terms of physicochemical properties and durability (Urban et al., 2018; Saghiri, 2024).

XRD analysis provided additional insight into the crystalline phase evolution of the tested sealers. The presence of Ca(OH)_2_ across all TSBS groups confirmed active hydration processes. Materials such as AHPBC and CS displayed relatively stable crystallinity over time, supporting the notion that their internal structure remains intact despite moderate solubility. In contrast, BRCS showed a marked decline in crystallinity at 6 months, which may be attributed to the conversion of calcium hydroxide to calcium carbonate in the presence of CO_2_, accompanied by decalcification of the calcium silicate hydrate (C–S–H) phase (Flores-Ledesma et al., 2020). Such alterations can disrupt the internal microstructure, leading to reduced crystallinity and potential weakening of the material (Abu-Zeid et al., 2024).

The MTAF exhibited strong initial crystallinity but substantially decreased after 6 months, with XRD patterns indicating the presence of unreacted tricalcium silicate (C_3_S) and reduced quantities of hydrated phases. These findings are consistent with the incomplete setting reactions reported in earlier studies (Abu Zeid & Edrees, 2022). The reduction in crystallinity at later stages may help explain the higher solubility observed for MTAF, as insufficiently hydrated material remains more susceptible to dissolution.

AHP demonstrated the most consistent crystallinity pattern among all groups, characterized by prominent peaks for calcium tungstate and zirconium oxide. These findings align with the material’s recognized stability and minimal solubility and further support the observation that epoxy resin–based sealers undergo negligible structural degradation during aging.

Although the present study provides valuable insight into long-term material behaviour, certain limitations must be acknowledged. Gravimetric solubility measurements using standardized discs do not fully replicate the complex root canal environment, where dentin interactions, moisture gradients, and functional stresses may alter material performance. Micro-CT–based volumetric analysis may offer more clinically relevant solubility data. Additionally, *in vivo* influences—such as enzymatic activity, inflammatory mediators, and cyclic loading—were not captured in this *in vitro* model.

Future investigations should aim to incorporate dentin-sealer interactions, advanced imaging techniques, and extended *in vivo* studies to better approximate clinical conditions. Further evaluation of particle size distribution, viscosity, setting dynamics, and radiopacity may also help clarify performance differences among contemporary sealers.

Despite these limitations, the results clearly demonstrate that the long-term stability of root canal sealers vary considerably across material classes. TSBS formulations offer valuable bioactivity, however they may exhibit increased solubility and greater structural changes over time. In contrast, epoxy resin–based AHP maintains superior stability, emphasizing the importance of careful material selection when long-term sealing capability is a priority.

## Conclusion

This study demonstrated clear differences in the long-term solubility and crystalline phase behaviour of the five evaluated root canal sealers when immersed in PBS over 24 h, 28 days, and 6 months. TSBSs showed varying degrees of solubility and structural changes, with BRCS and MTAF exhibiting the greatest degradation over time. AHPBC and CS maintained comparatively stable crystalline profiles, although both demonstrated moderate solubility. AHP consistently showed the lowest solubility and the most stable crystalline structure, reinforcing its reputation for long-term physicochemical reliability.

These findings highlight the importance of considering both solubility and crystallinity when selecting a sealer, as excessive dissolution and structural instability may compromise long-term sealing ability. While TSBSs provide the benefit of bioactivity, this must be balanced against their potential for increased solubility.

## Additional Information and Declarations

### Data Availability

The following information was supplied regarding data availability: The raw data on solubility analysis of root canal sealers is available at figshare: Shetty, Dr. Karthik (2025). Raw data set of solubility analysis of the 5 root canal sealers. figshare. Dataset. https://doi.org/10.6084/m9.figshare.30354082.v1.
